# Relations Among Maternal Life Satisfaction, Shared Activities, and Child Well-Being

**DOI:** 10.3389/fpsyg.2018.00739

**Published:** 2018-05-23

**Authors:** Nina Richter, Rebecca Bondü, C. Katharina Spiess, Gert G. Wagner, Gisela Trommsdorff

**Affiliations:** ^1^Developmental Psychology and Cross-Cultural Psychology, Department of Psychology, University of Konstanz, Konstanz, Germany; ^2^Psychologische Hochschule Berlin, Berlin, Germany; ^3^German Institute for Economic Research, Berlin, Germany; ^4^School of Business and Economics, Free University of Berlin, Berlin, Germany; ^5^Max Planck Institute for Human Development, Berlin, Germany

**Keywords:** self-regulation, maternal life satisfaction, well-being, prosocial behavior, German Socio Economic Panel Study, SOEP

## Abstract

Maternal well-being is assumed to be associated with well-being of individual family members, optimal parenting practices, and positive developmental outcomes for children. The objective of this study was to examine the interplay between maternal well-being, parent-child activities, and the well-being of 5- to 7-year-old children. In a sample of *N* = 291 mother-child dyads, maternal life satisfaction, the frequency of shared parent-child activities, as well as children’s self-regulation, prosocial behavior, and receptive vocabulary were assessed using several methods. Data were collected in a special study of the Socio-Economic Panel Study (SOEP), a representative longitudinal survey of private households in Germany. Using structural equation modeling, significant positive direct and indirect relations between maternal life satisfaction, frequency of shared parent-child activities, children’s self-regulation, prosocial behavior, and receptive vocabulary were found. The more satisfied the mother was, the more she shared activities with her child and the more the child acted prosocially. Furthermore, the higher the frequency of shared parent-child activities, the higher the child scored in all three analyzed indicators of children’s well-being: self-regulation, prosocial behavior, and receptive vocabulary. The current study supports the assumption of maternal well-being as the basis of positive parenting practices and child well-being.

## Introduction

Children’s well-being not only depends on their own emotional, social, and cognitive functioning, but also on environmental factors. Amongst these, familial factors, such as maternal well-being are particularly important and influential. Maternal well-being may possibly affect children’s outcomes via its impact on parenting, for example. In her Theory of Change, Newland (2015) defines “family well-being” as the foundation of “developmental parenting” and “child well-being.” Hence, how parents treat their children and are involved in shared activities impacts children’s development.

Maternal characteristics, such as well-being and life satisfaction, may also affect children’s emotional and behavioral health. In line with Newland’s (2015) Theory of Change, Agache (2017) also suggests the advantage of studying child well-being in relation to family well-being and various parenting factors. By emphasizing the cultural context as well as the person-culture fit, Trommsdorff (2009, 2018) states that interacting environmental factors impact individual differences in self-regulation and well-being. These factors include socio-economic, cultural, family, and school related factors, as well as biological predispositions. Previous research tested various meaningful pathways of Newland’s Theory of Change across several populations and cultures (Trommsdorff and Cole, 2011; Trommsdorff, 2012; Jaramillo et al., 2017) as well as connections, whether direct or indirect, from family to child well-being. In most of these studies, parenting and thus, child well-being are negatively affected by maternal depression, anxiety, distress, or other factors that threaten maternal well-being (Kam et al., 2011; Gündüz et al., 2014). The potential links of maternal well-being and life satisfaction with specific parenting practices, such as the involvement in shared activities, as well as child well-being and positive psychosocial adjustment have been far less researched. According to Brajša-Žganec and Hanzec (2014) analyzing positive factors that contribute to positive outcomes may help in developing methods aiming to promote these factors, whereas identifying and understanding negative factors may contribute to their prevention. Therefore, the present study sought to simultaneously examine representative positive components of all three Theory of Change model elements: maternal life satisfaction, shared parent-child activities, child’s self-regulation, prosocial behavior, and receptive vocabulary.

Maternal life satisfaction is of fundamental importance in family well-being (Headey et al., 2014) and one central component of parental mental health in Newland’s Theory of Change (Newland, 2015). Links between maternal well-being and child well-being are well-established (Holte et al., 2014); as Carter (2010, p. 6) states “a happy parent is a great role model.” According to Berger and Spiess (2011), the effect of maternal life satisfaction on a mother’s preschool child’s prosocial behavior across 159 mother-child dyads was “amazingly high.” Further, the authors found significant positive relations between maternal life satisfaction and the verbal and motor skills of 2- to 3-year-old infants. Similarly, Brajša-Žganec and Hanzec (2014) reported that life satisfaction of mothers is correlated both positively with prosocial behavior and negatively with conduct problems of their children at preschool age. Taylor and Conger (2017) concluded that boosting the well-being of single mothers, for example via group-based interventions that strengthen their social support networks, also increased the well-being of their children. Moreover, long-term effects were reported: Findings of Totsika et al. (2013) suggested that lower maternal life satisfaction is a risk factor for child behavior problems 4 years later. However, research on the effects of positive components of well-being (e.g., life satisfaction and happiness), on children’s development is rather rare. In contrast, interrelations between maternal depression or distress and children’s outcomes have often been described (see Smith, 2004, for a review). Depressive symptoms and parenting stress in mothers were associated with more emotional and behavioral difficulties of their children as well as problems in children’s self-regulation (Silk et al., 2006; Jackson et al., 2013; Huhtala et al., 2014). Likewise, Goodman and Garber (2017) reviewed intervention and therapy programs that successfully improved mental health in children by treating maternal depression.

Maternal well-being is critical not only to child development and well-being but also to high-quality parenting. High levels of maternal well-being form the basis for positive parenting practices, such as shared parent-child activities, because for mothers struggling with well-being difficulties it might be harder to be involved in activities with their children. Indeed, mothers who scored high in distress (i.e., symptoms of stress, anxiety, depression, and fatigue) were less engaged in shared play and learning activities with their children (Giallo et al., 2013). Similarly, Reissland et al. (2003) reported a significant relation between maternal mental health and the quality of shared activities. The authors found that maternal depression was related to the quality of a mother’s reading and speaking during a picture-book session with her infant. In contrast, Berger and Spiess (2011) did not find a relation between maternal life satisfaction and positive parenting practices as indicated by the frequency of shared activities.

The discussion of child well-being is characterized by definitional and conceptual confusion (Holte et al., 2014). Often, child well-being is not unraveled from terms such as “quality of life,” “life satisfaction,” and “happiness”; if not sometimes used synonymously. Still “it is not clear whether [these terms] focus on unique or on different phenomena, and whether they are related to the same underlying dimension” (Trommsdorff, 2018, p. 162). Moreover, child well-being has different meanings for different individuals, groups, and societies because it includes both objective factors and subjective evaluations (for more details, see Trommsdorff, 2018). Moore et al. (2012) stated that most indices of child well-being refer to negative aspects of development while positive outcomes were overlooked. Newland (2014) conceptualizes child well-being as comprising multidimensional positive child outcomes including five components: physical health, mental health, self-regulation, social competence, and cognitive competence. Following this definition of child well-being, the present paper focusses on three of its indicators: self-regulation, prosocial behavior (as an indicator of social competence), and language abilities (as an indicator of cognitive competence).

Self-regulation is the motivation and “ability of individuals to adjust their cognition, emotion, and behavior in order to meet both intrinsic and extrinsic demands” (McClelland et al., 2010; Edossa et al., 2017, p. 1). Although the complex multidimensional construct of self-regulation is a central topic in psychology today, a confusing mixture of concepts and definitions exists (for a detailed discussion, see Eisenberg, 2017; Nigg, 2017). Self-regulation is a crucial predictor of adjustment, maladjustment, and quality of social functioning in children and adolescents (Eisenberg et al., 2016, 2017). Furthermore, adequate self-regulation is known to predict an array of positive outcomes throughout the entire life span: not only are optimally self-regulated children more likely to be successful, healthy, wealthy, and stress tolerant, but they are less likely to be arrested or abuse substances later in life, irrespective of their intelligence (Ayduk et al., 2000; Caspi et al., 2003; Duckworth and Seligman, 2005; Moffitt et al., 2011, 2013). Because successful self-regulation is important for various positive developmental outcomes (Casey et al., 2011; Spinrad and Eisenberg, 2015), some even consider it to be an essential component of child well-being itself (Newland, 2014).

Various studies indicate interacting relationships between the different components of child well-being. Regarding the links between self-regulation and prosocial behavior, children capable of self-regulating their behavior have been shown not only to be more liked but also to demonstrate more prosocial behavior and social competencies than their peers, even years later as adolescents (Mischel et al., 1988; Eisenberg et al., 2004; Eisenberg et al., 2006; McClelland and Cameron, 2011; Eisenberg et al., 2014; Eisenberg, 2015; Flook et al., 2015). Furthermore, greater self-regulation skills of young adolescents have been shown to relate to a lesser decline of prosocial behavior during adolescence (Luengo Kanacri et al., 2013). Hence, adequate self-regulation positively impacts children’s social development. Regarding the links between self-regulation and verbal abilities, children’s successful self-regulation has been positively associated with language skills (von Salisch et al., 2015) and success in school (McClelland and Cameron, 2011; Allan et al., 2014; McClelland et al., 2014; Weis et al., 2016), even after controlling for socio-economic status (Edossa et al., 2017). Suchodoletz et al. (2009) demonstrated that the capacity to self-regulate behavior during kindergarten was related to reading and writing skills in school in a one-year follow-up. Finally, relationships between children’s cognitive and social competencies are often found (e.g., Cassidy et al., 2003). For instance, children’s verbal competencies at 2 years of age positively predicted their prosocial behavior at age four (Ensor et al., 2011). Hence, interrelations of the three components of well-being as examined in the present study are well-established. Self-regulation, however, may not ‘only’ be considered an aspect of well-being. Most often, self-regulation is considered to be a basic and central social-cognitive competence that reflects important aspects of executive functions, such as the ability to inhibit undesired behavior and to flexibly respond to the demands of the present situation. Thus, self-regulation may also be considered an important precursor of both social success and cognitive competencies. This view is represented in the studies cited above, which mainly used self-regulation as a predictor of various developmental outcomes; it is also the view we adopted in the present study.

In general, families and, in particular, mother-child relationships are of fundamental importance in child well-being. The benefits of positive parenting behavior, such as maternal warmth and sensitivity, on behavioral and emotional health as well as self-regulation abilities of children have long been established (Sénéchal and Lefevre, 2002; Eisenberg et al., 2005; Weinstein, 2005; Suchodoletz et al., 2011; Baker and Hoerger, 2012; Razza and Raymond, 2013; Russell et al., 2013; Brajša-Žganec and Hanzec, 2014; Houltberg et al., 2014; Davis et al., 2016). However, shared time in educational and enriching activities is also important for children’s development and well-being. A multitude of studies on social learning and socialization suggest that parent-child bonds provide opportunities for social skill learning by generating cognitive schemas that prove beneficial to interactions with peers in later life as well as contribute emotional assets for social exploration (Hartup, 1989; Eccles, 1999). Hence, enjoying and engaging in shared parent-child activities that require and train different forms of social, cognitive, and emotional competencies (e.g., give-and-take, turn-taking, and providing the child with new information) may support children’s self-regulation, cognitive, and socio-emotional development. Indeed, in a longitudinal study of over 3000 European children Sylva et al. (2004) demonstrated that parent’s engagement in shared activities (e.g., reading books, teaching songs, painting, playing with letters and numbers, creating opportunities to play with peers, and visiting libraries) was associated with positive behavioral, social and intellectual outcomes in their children 3 years later at age 6–7. The authors concluded: “For all children, the quality of the home learning environment is more important for intellectual and social development than parental occupation, education or income. What parents do is more important than who parents are.” (Sylva et al., 2004, p. 1). Similarly, Tudge et al. (2003) reported that children’s academic achievement depended on whether their parents provided educational toys, answered their questions, and shared conversations about their children’s experiences. Hsin (2009) brought into consideration that the positive results of parents’ time with their children largely depend on their ability to provide cognitive stimulation and verbal engagement. Hence, shared everyday activities and experiences provided by parents (e.g., book reading, talking, music activities, helping with homework, and eating meals together) were significantly associated with measures of children’s vocabulary, broad reading scores, numeracy, self-regulation, prosocial skills, and externalizing behavior (Fiorini and Keane, 2014; Hsin and Felfe, 2014; Raley, 2014; Williams et al., 2015; Fomby and Musick, 2017; Niklas and Schneider, 2017). Emphasizing the unique contributions of shared parent-child activities on child well-being, Katcher (2014) concluded that adolescents participating in parent-child activities showed lower levels of depression through a better quality of parent-child relationship. Moreover, shared activities with parents, like family meals, were negatively related to adolescent depression, independent of the contribution of warm parenting styles and relationship quality. In line, Carter (2010) suggests eating dinner together in the family as one substantial daily activity that improves the well-being of the family and child. Consequently, on the one hand, the quality of parenting practices as well as a caring and warm parent is substantial for child well-being. On the other hand, the parent-child “quality time” spent together such as enjoying and engaging in shared activities, is crucial for children’s self-regulation, cognitive, emotional, and social development (Hastings et al., 2007).

**FIGURE 1:**
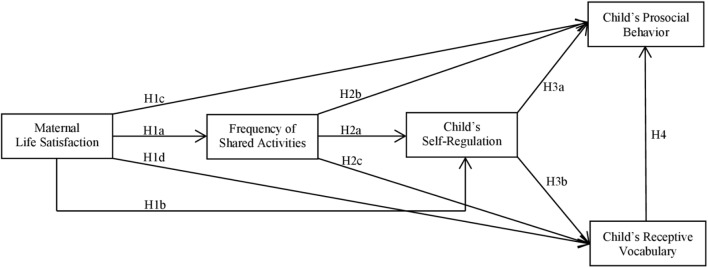
Well-being of family and child: Model of hypothesized relationships between maternal life satisfaction, frequency of shared parent-child activities as well as self-regulation, prosocial behavior and receptive vocabulary.

The present study builds on previous work by investigating the relationships between self-regulation, social competencies, and verbal abilities by identifying familial factors that may promote these components. As shown, abundant research tested the associations and effects between single aspects of the Theory of Change or mediator models. However, the role and impact of positive components (e.g., life satisfaction) has been far less researched. Consequently, relying on a dataset from the SOEP, our objective was to address this perceived gap by analyzing positive factors of all three components of the Theory of Change (Newland, 2015): maternal life satisfaction, shared parent-child activities, child self-regulation, prosocial behavior, and verbal abilities. In our study, we shifted the focus of interest from extensively studied negative factors (e.g., maternal depression, anxiety, and distress) to positive factors of well-being and development. We put a special focus on child self-regulation as it is an essential component of child well-being and a motor for further positive outcomes. In addition, maternal well-being is a fundamental component of family well-being (Headey et al., 2014) and in the present study it is related to frequent shared parent-child activities; one example of positive parenting practices. In turn, this is associated with successful self-regulation, prosocial behavior, and receptive vocabulary, central components of child well-being. Following Newland’s Theory of Change, we hypothesize that maternal satisfaction with life is positively related to the frequency of shared activities as well as child well-being. See **Figure 1** for a model of the hypothesized relationships. The more satisfied the mother is, the more capacity she has to share activities with her child and the higher the child’s self-regulatory skills, his/her prosocial behavior, and receptive vocabulary (hypotheses H1a/H1b/H1c/H1d). The higher the frequency of shared parent-child activities, the higher the child’s self-regulation, prosocial behavior, and receptive vocabulary (hypotheses H2a/H2b/H2c). In line with previous research on the positive outcomes of self-regulation, we further hypothesize that self-regulating skills are positively connected to prosocial behavior and receptive vocabulary. We expect children who are more capable of self-regulating their behavior to act more prosocial according to their mothers (hypothesis H3a) and to have a greater receptive vocabulary (hypothesis H3b). Finally, we expect a positive relationship between receptive vocabulary and prosocial behavior (hypothesis H4).

## Material and Methods

### Participants

Analyses were based on a special study of the German Socio-Economic Panel Study (SOEP; Wagner et al., 2007). The SOEP is a representative longitudinal survey of private households in Germany since 1984. Our study includes 291 mother-child dyads (TNS Infratest Sozialforschung, 2008). Children were aged between 5 and 7 years (*M* = 6 years 1 month) and 54.6% were male (*n* = 159). The mean age of the mothers was 36 years (*SD* = 5). From this sample, 40 mothers (13.8%) had a university degree, 228 mothers (78.6%) completed vocational training, 22 mothers (7.6%) had not completed vocational training, and one mother did not answer this question. The mean monthly net household income was 2.391 Euro (*SD* = 1029) and thus lay bellow the German average of 2.914 Euro net income per household per month in 2008 (Kott and Behrends, 2011).

### Mother Related Measures

#### Maternal Life Satisfaction

Maternal life satisfaction was measured via the single item, “How satisfied are you with your life, all things considered?” Response options ranged from 0 (*completely dissatisfied*) to 10 (*completely satisfied*). Single-item measures of life satisfaction have reasonable validity and correlate moderately with other measures of well-being and positive affect including questionnaires, written interviews, informant reports, and measures of daily affect (Sandvik et al., 1993). They are widely used in survey based psychological research (e.g., Schimmack et al., 2008).

#### Frequency of Shared Activities

The frequency of shared activities was assessed using eleven items based on the question, “How often have you or your child’s main caregiver engaged in the following activities together with your child in the last 14 days? (1) singing children’s songs; (2) going for walks outdoors; (3) painting or doing crafts; (4) playing cards, board games, or other games together; (5) going to the playground; (6) visiting other families with children; (7) going shopping with the child; (8) watching TV or videos together; (9) playing computer or Internet games together; (10) going to children’s theater, the circus, museums, exhibitions, or similar; (11) reading or telling stories in German.” The 4-point scale ranged from 1 *(not at all)* to 4 *(daily)*. The variable represents the mean score of all eleven items. Higher scores indicated more shared parent-child activities, as perceived by the mother.

### Child Related Measures

#### Self-Regulation

Children’s self-regulation was measured by monitoring their behavior during the interview using a candy delay task. Organic gummi bears were offered from an already opened bag on the table. The child had to resist the temptation to eat the candies until the interview with the mother was finished in order to get the opened bag of gummi bears along with a second bag. If the child did not resist, they would only receive this one, opened, bag of gummi bears and not a second bag. The child’s understanding of the contingency was tested and ensured by repeating the instructions if necessary. Mothers were instructed not to influence their children in their behavior. On average, the interview took 48 min, with 79.0% of the children (*n* = 230) resisting the temptation to eat the candy during the interview, thus showing self-regulating behavior (1 = *ate candy during the interview, got 1 bag*; 2 = *didn’t eat candy during the interview, got 2 bags*). There were no significant sex differences.

#### Prosocial Behavior

To measure the child’s prosocial behavior, mothers assessed the accordant 5-item subscale of the Strengths and Difficulties Questionnaire (SDQ; e.g., “My child is thoughtful.”; Goodman, 1997; Woerner et al., 2002). Response options ranged from 0 *(does not apply)* to 2 *(applies completely)*. See **Table 1** for the mean, standard deviation, and Cronbach’s alpha of the single variable score (mean of all 5 items).

**Table 1:** Bivariate Correlations, means, standard deviations, and Cronbach’s alphas for the study variables.

Variable	mLS	SA	SR	PB	RV
Maternal life satisfaction	–	**0.25**	0.03	**0.30**	0.06
Frequency of shared activities		–	0.11	**0.26**	**0.19**
Self-regulation			–	**0.28**	**0.13**
Prosocial behavior				–	**0.14**
Receptive vocabulary					–
M (N = 291)	7.29	2.48	–	7.89	50.07
SD (N = 291)	1.88	0.37	–	1.84	7.54
Cronbach’s α	–	0.63	–	0.67	0.90


*Significant correlations (*p* < 0.05) are printed in bold. mLS = Maternal life satisfaction, SA = Frequency of shared activities, SR = Self-regulation, PB = Prosocial behavior, RV = Receptive vocabulary.*

#### Receptive Vocabulary

Receptive vocabulary was measured with a passive verbal test; a shortened version of the Peabody Picture Vocabulary Test Revised (PPVT-R; Dunn and Dunn, 1981). The test consisted of 61 stimulus words and 61 corresponding image plates showing four black-and-white drawings. The child had to figure out which of the four drawings best represented the meaning of the corresponding stimulus word. The single variable score describes the sum of right answers and was used for the correlation analyses (see **Table 1**).

### Control Variables

Analyses were controlled for the child’s age and their sex (see section “Participants” for details). Furthermore, the liking of gummi bears measured on a 4-point scale ranging from 1 (*likes them not at all*) to 4 (*likes gummi bears very much*) was used as a control variable (*M* = 3.61, *SD* = 0.65). Additionally, we controlled for having siblings (1 = *yes*; 2 = *no*) and attending a day-care center/school (1 = *day-care center*; 2 = *school*; 3 = *none*). In the present sample, 231 children (79.4%) had siblings and 217 (74.6%) visited a day-care center, whereas 71 (24.4%) attended school (3 (1%) did neither visit day care nor school). Furthermore, maternal education (we differed between no school-leaving qualification, secondary modern school-leaving certificate, intermediate school-leaving certificate, and high standard school-leaving certificate) as well as mother’s verbal abilities were used as maternally related control variables. Mother’s verbal abilities were assessed with 36 items of the multiple-choice vocabulary intelligence test (MWT; Lehrl, 2005). The MWT measures the vocabulary and thereby provides an approximate estimate of (crystallized) intelligence. The task is to pick the one out of five phonetically plausible combinations of letters that form an existing German term. In this sample, the mothers correctly answered 30 questions, on average (*M* = 29.67, *SD* = 3.73; *Cronbach’s alpha* = 0.80).

### Procedure

Mothers and children were invited to take part in the special SOEP study, “Your Child at Preschool Age” in 2007/08 (TNS Infratest Sozialforschung, 2008). This study, conducted by the TNS Infratest Sozialforschung (now: Kantar Public) field work organization, assessed, to the best of our knowledge for the first time, competencies and behavior variables of preschool children under survey conditions, which means in a household context (for other studies based on this data, see e.g., Bartling et al., 2010; Drobetz et al., 2012; Kosse and Pfeiffer, 2012). The computer assisted personal interview encompassed questions about mother and child. In addition, both mothers and their children completed a verbal ability test. To assess children’s self-regulation, the aforementioned observational measure was used: during the interview the child sat next to the mother and the child’s self-regulatory skills were directly observed. Ethical permission was provided by the Scientific Advisory Board of DIW Berlin. Participation of mother and child was entirely voluntary. Mothers were informed about the procedure and informed consent was obtained. During the assessment of the child related measures mothers were present. Interviewers were equipped with detailed instructions including word for word formulations as well as comments to appropriate reactions, in particular situations to reduce potential sources of errors and to make sure reliable and valid data was collected.

Mothers and children widely accepted the test material, which was extensively adjusted for survey usage. Children were highly motivated and attentive during the testing procedure; over 96% of interviewers answered the question concerning a high level of children’s motivation with *fully applies* or *rather applies*.

### Statistical Analyses

**FIGURE 2:**
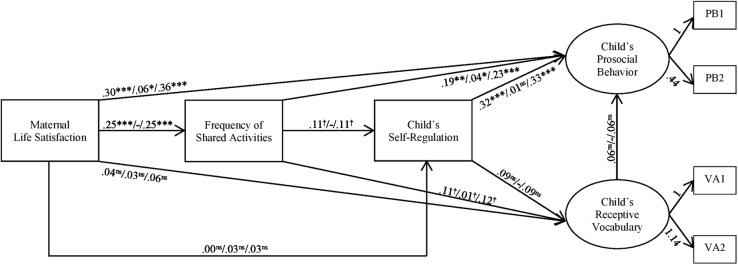
Structural model predicting the latent factors prosocial behavior and receptive vocabulary from the manifest variables self-regulation, frequency of shared parent-child activities, and maternal life satisfaction without control variables. Printed path estimates are standardized β-coefficients in the following order: direct effect/indirect effect/total effect; ^†^*p <* 0.1, *^∗^p* < 0.05, *^∗∗^p* < 0.01, ^∗∗∗^*p* < 0.001. *N* = 291 mother-child dyads.

Structural equation modeling was used for data analyses using the ‘sem’ command of the statistic software Stata (StataCorp, 2013). Using structural equation modeling, we were able to simultaneously determine direct and indirect effects between all the mentioned variables and to consider multiple outcome measures simultaneously. Two latent variables, namely prosocial behavior and receptive vocabulary, as well as three observed variables, namely maternal life satisfaction, frequency of shared activities, and self-regulation, formed the structural model. Latent variables have the advantage of being adjusted measurement error. The two latent variables were each defined by two parcels summing up either the prosocial behavior items (PB1 = Item 1 + 2 + 3; PB2 = Item 4 + 5; see **Figure 2**) or the receptive vocabulary test items (RV1 = all pair items between 2 and 60; RV2 = all impair items, between 1 and 61). Defining parcels results in more stable latent variables for structural equation analyses than does the use of single items (Widaman et al., 2010). The loadings of the first indicators (RV1 and PB1) were fixed to 1.

To assess model fit the *likelihood ratio test* (χ*^2^*), *root mean square error of approximation* (RMSEA), *comparative fit index* (CFI), and *standardized root mean square residual* (SRMR) were considered. The following cut-off values were used to indicate an acceptable model fit: χ*^2^*, *p* > 0.05; RMSEA < 0.05; CFI > 0.95; and SRMR < 0.08 (Hu and Bentler, 1999). Using the ‘estat eqgof’ command, we further computed the *R*^2^ for the dependent variables prosocial behavior and receptive vocabulary.

## Results

Correlations, means, standard deviations, and internal reliability scores (Cronbach’s alphas) for the study variables of mothers and their children are presented in **Table 1**. As shown, there were positive correlations between prosocial behavior and all other studied variables: maternal life satisfaction, frequency of shared parent-child activities, self-regulation (0.26 ≤*r* ≥ 0.30, *p* < 0.001), and receptive vocabulary (*r* = 0.14, *p* = 0.016). Additionally receptive vocabulary was positively correlated to the frequency of shared activities and self-regulation (0.13 ≤*r* ≥ 0.19, *p* < 0.05). Further, the correlation between maternal life satisfaction and the frequency of shared activities was significant (*r* = 0.25, *p* < 0.001). In terms of the control variables mother’s verbal abilities significantly correlated with mother’s satisfaction with life (*r* = 0.19, *p* = 0.001) as well as children’s verbal abilities (*r* = 0.20, *p* < 0.001). Furthermore, children who already visited school scored higher in the receptive vocabulary test and shared less activities with their parents compared to children attending daycare. In general, both mothers and children performed well in the vocabulary tests as compared to other samples in Germany.

### Pathways Between Maternal Life Satisfaction, Shared Activities and Child Well-Being

**FIGURE 3:**
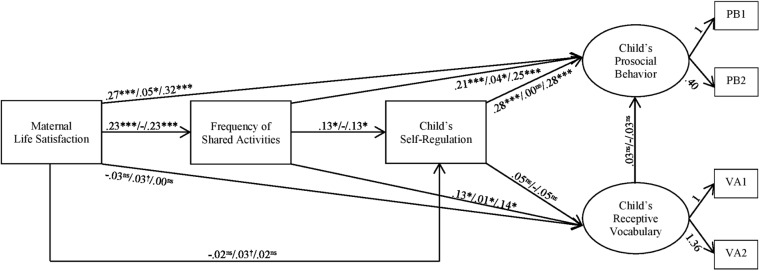
Structural model predicting the latent factors prosocial behavior and receptive vocabulary from the manifest variables self-regulation, frequency of shared parent-child activities, and maternal life satisfaction. All equations are controlled for child’s age and sex. selective equations are controlled for child’s liking for gummi bears and having siblings (Hlb, H2a). visiting day-care/school (H1d, H2c, H3b), mothers verbal abilities (HIa, H1d, H2c, H3b) and mother’s education (Hla, Hlc, Hid, H2b, H2c, H3a, H3b). Printed path estimates are standardized β-coefficients in the following order: direct effect/indirect effect/total effect; ^†^*p <* 0.1, *^∗^p* < 0.05, *^∗∗^p* < 0.01, ^∗∗∗^*p* < 0.001. *N* = 291 mother-child dyads.

As shown in **Figures 2**, **3**, both the uncontrolled and controlled model, had a good to very good model fit, depending on the fit statistic; χ*^2^* (7, *n* = 291) = 6.19, *p* = 0.52; RMSEA = 0.00; CFI = 1.00; and SRMR = 0.01 (model without control variables) and χ*^2^* (32, *n* = 291) = 34.52, *p* = 0.35; RMSEA = 0.02; CFI = 0.99; and SRMR = 0.03 (model with control variables), respectively. Due to negligible differences between the results of the uncontrolled and the controlled model, only results of the controlled model are discussed (see **Figure 3**).

Maternal life satisfaction was significantly directly related to the frequency of shared parent-child activities (β = 0.23, *p* < 0.001) and prosocial behavior (β = 0.32, *p* < 0.001), see **Figure 3**. The more satisfied the mother was, the more often she shared activities with her child and the higher she evaluated her child’s prosocial behavior. No direct relationships between maternal life satisfaction and child’s self-regulation or verbal abilities were found. Maternal life satisfaction also showed a significant indirect link with the child’s prosocial behavior (β = 0.05, *p* = 0.043) through the frequency of shared activities. There were additional marginally significant indirect effects on child’s self-regulation (β = 0.03, *p* = 0.051) and receptive vocabulary (β = 0.03, *p* = 0.072) through the frequency of shared activities.

The frequency of shared activities was significantly directly related to self-regulation (β = 0.13, *p* = 0.026), prosocial behavior (β = 0.25, *p* < 0.001), and verbal abilities (β = 0.14, *p* = 0.036). The more often mother and child engaged in shared activities, the higher the child’s well-being, that is, its self-regulation, prosocial behavior, and verbal abilities. In addition, the frequency of shared activities was indirectly connected to prosocial behavior (β = 0.04, *p* = 0.015) and verbal abilities (β = 0.01, *p* = 0.026) via the child’s self-regulation abilities. Thus, results were in line with hypotheses H1a/H1b/H1c/H1d, H2a/H2b/H2c due to direct and/or indirect relationships between the variables.

### The Role of Self-Regulation for Child Well-Being

As expected in H3a, self-regulation was significantly and positively related directly to prosocial behavior (β = 0.28, *p* < 0.001). Children with higher self-regulatory skills were rated as more prosocial by their mothers. However, contrary to hypothesis H3b, self-regulatory skills were not related to verbal abilities, that is, receptive vocabulary (β = 0.05, *p* = 0.391). Moreover, contrary to H4, children’s receptive vocabulary did not predict their prosocial behavior (β = 0.03, *p* = 0.682).

The *R*^2^ applying to the variable receptive vocabulary was 0.112 and of prosocial behavior 0.296, so 11.2 and 29.6%, respectively, of variance are explained by the model.

## Discussion

The present study identified positive direct and indirect relationships between maternal well-being, specific parenting practices, and child well-being by using different methods and informants. Results revealed significant positive relations between maternal life satisfaction and the frequency of shared everyday activities and experiences as well as child’s prosocial behavior. The more satisfied the mother was with life in general, the more she was involved in shared parent-child activities. Further, children of satisfied mothers scored higher in prosocial behavior compared to children of less satisfied mothers. This is consistent with well-established results showing that both parental well-being and optimal parenting practices promote children’s prosocial behavior and social skills (e.g., twin study by Eisenberg et al., 2006; Neece and Baker, 2008). Similarly, in a meta-analysis Trivette et al. (2010) reported that parental well-being directly and positively affected both parent-child interaction (measured by the frequency of parent-child engagement in 24 different games) and child development. Additionally, in the present study, maternal life satisfaction was indirectly marginally significantly related to child’s self-regulation and verbal abilities with the frequency of shared activities as mediator. More satisfied mothers were more involved in activities with their children; as expected; children showed higher self-regulatory and receptive vocabulary test skills than children of less satisfied mothers. We therefore infer that maternal life satisfaction plays a significant role for maternal involvement in child-oriented activities and several positive developmental outcomes. Further, our results show a positive relationship between easy to realize shared everyday parent-child activities with 5- to 7-year-old children and child well-being. This is in line with research highlighting and promoting the various positive effects of shared activities, e.g., reading and telling stories to children foster their language development (Whitehurst et al., 1988; Swanson et al., 2011). Taken together, our results are in accordance with Newland’s Theory of Change. We extended previous research by simultaneously analyzing interrelations of all three model elements. There were positive relations between maternal well-being, parental behavior, and child well-being. In the future, knowledge may help in developing methods and programs aiming to promote these factors (Brajša-Žganec and Hanzec, 2014).

Our results are also in line with previous research that outlined a positive and meaningful effect of self-regulation on prosocial behavior in children (Carlo et al., 2012; Eisenberg, 2015). In this study, there was a positive relation of children’s self-regulatory skills and their social skills as rated by their mothers. Contrary to our expectation and to the literature, we did not find an association between self-regulation and language abilities, that is receptive vocabulary. This might be due to the fact, that in our sample, the frequency of shared activities (β = 0.14, *p* = 0.036), the child’s age (β = 0.16, *p* = 0.032), and the verbal abilities of the mother (β = 0.28, *p* < 0.001) were stronger predictors of children’s receptive vocabulary than their self-regulatory skills. Moreover, contrary to previous research and our hypothesis H4, children’s receptive vocabulary did not predict their prosocial behavior. This might be due to differences in the age of the children in this study and the studies cited above. For example, Cassidy et al. (2003) reported associations between verbal competencies and prosocial behavior in children aged three to five compared to the 5-to 7-year-olds in the present sample. Moreover, methodological differences should be mentioned: Ensor et al. (2011) came to the result that emotion understanding mediated the effect of verbal abilities at age 2 on prosocial behavior at age 4.

### Limitations and Avenues for Further Research

A main limitation of this study is the cross-sectional nature of the data. Thus, only assumptions about the directions of the effects are feasible. It is possible that the demand characteristics of a child with behavior problems affect maternal well-being and parenting practices to a significant degree (Krahé et al., 2015). Indeed, evidence from transactional studies support bidirectional parent-child influences as well as reciprocal relations between parental well-being, parenting behavior, and children’s behavior problems (e.g., Ciciolla et al., 2014; Eisenberg et al., 2015). Musick et al. (2016) reported that parents scored higher in subjective well-being during activities with children than without, indicating that shared time, activities, and experiences with children may positively affect parents’ well-being and life satisfaction. Likewise, Elam et al. (2017) found that children’s externalizing behavior in middle childhood predicted lower levels of positive parenting in early adolescence. These studies are in line with Morris’s model of the development of emotion regulation in the familial context (Morris et al., 2007), which also stipulates bidirectional pathways. The model states that both parental and child characteristics influence the familial socialization processes, and past interactional experiences impact further socialization. This implies that the results of the present study, on the one hand, underpinned the relations between family well-being and child well-being. On the other hand, causal relations could not be tested.

A further limitation concerns the unexpectedly high proportion of children who self-regulated their behavior (79%) and successfully completed the candy delay task. Kidd et al. (2013) reported that children’s waiting times on delay of gratification tasks are moderated by beliefs about environmental reliability; children in the reliable environment waited significantly longer compared to their counterparts. As our data were assessed at home (in a most likely reliable environment), self-regulatory skills might have been high. Furthermore, consistent with previous studies on delay tasks (e.g., Murray and Kochanska, 2002) indicating that self-regulating skills increase with age, the result might be the consequence of the relatively high age of the children in our study with a mean of 6 years 1 month (*Min* = 62 months, *Max* = 85 months). In our sample, 117 children (40.2%) were 5 years old, 158 children (54.3%) were at age 6, and 16 children (5.5%) were 7 years old. 81 5-year-old children (69.2%) waited and did not eat the organic gummi bears during the interview in order to receive a second bag compared to 136 of the 6-year olds (86.1%), and 13 of the 7-year olds (81.3%). This difference between the age groups was significant (Fisher’s exact test: *p* = 0.003; the groups ‘age 5’ and ‘age 6’ differ significantly, with adjusted residuals > 3). The presence of the mother may have also influenced the results. Extensive preliminary considerations were performed to generate an adequate study design for a household situation and different experiments formed the basis for the finally chosen SOEP design (Mischel and Metzner, 1962; Arend et al., 1979; Mischel and Mischel, 1983). The following requirements were met by the adapted behavior experiment: the influence of confounding factors (e.g., influence of the mother) was minimized, easy and standardized administration through the trained interviewer, and practical applicability in a limited period of time in a household context.

The data of our study were assessed at the homes of the subjects within a survey framework. Specific surrounding conditions probably influenced the data assessment compared to a strictly standardized study environment and, therefore, might restrict their validity. Uncontrollable events due to other household members, telephone calls, social components (e.g., maternal presence during the test situation), or little breaks (e.g., so that the interviewer and the mother “could listen to what the child wanted to tell” them) are exemplary deviations from the predetermined procedure. Nevertheless, the number of interviews during which a serious disruption was reported was low (disturbances caused by a ringing phone or door-bell, additional persons, or pets in 3.8–8.9% of the cases, depending on the test).

Further limitations include the single item measure of maternal life satisfaction as well as the relatively low internal reliability for mother-reported prosocial behavior. Moreover, as noted above, the mean monthly net household income in this sample was 2.391 Euro (*SD* = 1029) and thus lay bellow the German average of 2.914 Euro net income per household per month in 2008 (Kott and Behrends, 2011). This may diminish the generalizability of the results. Last but not least, the strength of the significant correlations between the studied variables is generally weak (0.13 ≤*r* ≥ 0.30). This should be taken into consideration when interpreting the SEM results of this study; though associations reached statistical significance, they are merely small to moderate.

Taken together, our data are based on behavioral observations and psychological tests and were used for a multi-method and multi-informant cross-sectional study providing meaningful results largely in line with Newland’s Theory of Change Model.

## Conclusion

Despite some limitations, some major methodological and practical implications arise from the results.

### Methodological Implications

Results illustrate direct and indirect pathways through which maternal life satisfaction is associated with shared parent-child activities and child well-being. First, further representative longitudinal studies are needed to test the theoretical framework and the direction of its pathways. Second, future studies should take additional components (e.g., parental well-being, well-being of siblings) as well as person-context relational processes into consideration. Third, as the theoretical reference should be tested in its entirety across diverse samples, age groups, family constellations, and cultures, additional factors may be determined, which moderate or mediate the pathways outlined in this theoretical frame.

### Practical Implications

Clearly, the importance of child well-being cannot be overstated and the fundamental role of high maternal life satisfaction is impressive. This study confirms what multiple “mom-blogs” (e.g., Burke Harris, 2015) point out: Mothers must first mother themselves in order to truly mother their children well. In other words, if the mother herself is satisfied with life and feels looked after, she is able to look after the child. The way to a healthy and optimal developed child is through their mother’s well-being. Fortunately, many effective (home-based) intervention and prevention programs were designed to enhance maternal well-being and family functioning, thereby improving both parenting practices and child well-being (Beeber et al., 2004; Diamond et al., 2007; Groeneveld et al., 2016; Tanninen et al., 2016). These interventions and prevention programs address different familial constellations, at risk families, various age groups, as well as clinical and non-clinical groups (for current research on evidence-based interventions and parenting programs, see Luthar and Eisenberg, 2017). From an economic perspective, high quality interventions starting early in life have proven advantageous, due to high long-term socio-economic profits (for an overview and statements for policy advice, see German National Academy of Sciences Leopoldina, 2014). It is without question that the high pedagogical quality of these intervention and prevention programs should be ensured. In addition, there are prevention programs, which are not directly focused on family well-being but aimed at promoting shared activities such as playing, reading, and story-telling and, thereby, child well-being (Moore and Wade, 2003; Lange, 2014), such as the public health initiatives “Lesestart” in Germany^1^ or “Bookstart” in the UK^2^. The present study underscores the importance of promoting high-quality and effective intervention programs that pursue the goal of strengthening family resources for the well-being of mothers and children.

## Author Contributions

NR wrote the manuscript with support from GT, RB, CKS, and GGW. NR, GT, and RB analyzed and interpreted the data. GT, CKS, and GGW made substantial contributions to conception and design of the study as well as the acquisition of data. All authors provided critical feedback and gave final approval of the version to be published.

## Conflict of Interest Statement

The authors declare that the research was conducted in the absence of any commercial or financial relationships that could be construed as a potential conflict of interest. The reviewer MS and the handling Editor declared their shared affiliation.

## Footnotes

1 www.lesestart.de

2 www.booktrust.org.uk/supporting-you/families/our-programmes/bookstart/
